# Supplementary material for: Smooth Interpolating Curves with Local Control and Monotone Alternating Curvature
Source: Comput Graph Forum. 2022 Oct 6;41(5):25–38. doi: 10.1111/cgf.14600 (PMC9827861; doi:10.1111/cgf.14600)
Supplement: Supplementary file 1 — Supplement Material [file CGF-41-25-s001.zip › Local-Smooth-Interpolating-MonoCurvature/extern/clothoids/docs/api-matlab/program_listing_file_ClothoidCurve.m.html]

Program Listing for File ClothoidCurve.m — Clothoids v2.0.9

### Navigation

- index
- toc
- Clothoids »
- Program Listing for File ClothoidCurve.m

# Program Listing for File ClothoidCurve.m¶

↰ Return to documentation for file (`ClothoidCurve.m`)

```
classdef ClothoidCurve < CurveBase
  methods
    %> Create a new C++ class instance for the
    %> clothoid arc object
    %>
    %> **Usage:**
    %>
    %> \rst
    %> .. code-block:: matlab
    %>
    %>    ref = ClothoidCurve()
    %>    ref = ClothoidCurve( x0, y0, theta0, k0, dk, L )
    %>
    %> \endrst
    %>
    %> **On input:**
    %> - `x0`, `y0`: coordinate of initial point
    %> - `theta0`:   orientation of the clothoid at initial point
    %> - `k0`:       curvature of the clothoid at initial point
    %> - `dk`:       derivative of curvature respect to arclength
    %> - `L`:        length of curve from initial to final point
    %>
    %> **On output:**
    %>
    %> - `ref`: reference handle to the object instance
    %>
    function self = ClothoidCurve( varargin )
      self@CurveBase( 'ClothoidCurveMexWrapper' );
      self.objectHandle = ClothoidCurveMexWrapper( 'new', varargin{:} );
    end
    % - - - - - - - - - - - - - - - - - - - - - - - - - - - - - - - - - - -
    function str = is_type( ~ )
      str = 'ClothoidCurve';
    end
    % - - - - - - - - - - - - - - - - - - - - - - - - - - - - - - - - - - -
    %>
    %> Build the clothoid from known parameters
    %>
    %> **Usage:**
    %>
    %> \rst
    %> .. code-block:: matlab
    %>
    %>    ref.build( x0, y0, theta0, k0, dk, L )
    %>
    %> \endrst
    %>
    %> **On input:**
    %>
    %> - `x0`, `y0`: coordinate of initial point
    %> - `theta0`:   orientation of the clothoid at initial point
    %> - `k0`:       curvature of the clothoid at initial point
    %> - `dk`:       derivative of curvature respect to arclength
    %> - `L`:        length of curve from initial to final point
    %>
    function build( self, varargin )
      ClothoidCurveMexWrapper( 'build', self.objectHandle, varargin{:} );
    end
    % - - - - - - - - - - - - - - - - - - - - - - - - - - - - - - - - - - -
    %> Build the interpolating G1 clothoid arc
    %>
    %> **Usage:**
    %>
    %> \rst
    %> .. code-block:: matlab
    %>
    %>    ref.build_G1( x0, y0, theta0, x1, y1, theta1 )
    %>
    %> \endrst
    %>
    %> **On input:**
    %>
    %> - `x0`, `y0`: coordinate of initial point
    %> - `theta0`:   orientation of the clothoid at initial point
    %> - `x1`, `y1`: coordinate of final point
    %> - `theta1`:   orientation of the clothoid at final point
    %>
    function varargout = build_G1( self, x0, y0, theta0, x1, y1, theta1 )
      if nargout > 1
        [ varargout{1:nargout} ] = ClothoidCurveMexWrapper( ...
          'build_G1_D', self.objectHandle, x0, y0, theta0, x1, y1, theta1 ...
        );
      else
        [ varargout{1:nargout} ] = ClothoidCurveMexWrapper( ...
          'build_G1', self.objectHandle, x0, y0, theta0, x1, y1, theta1 ...
        );
      end
    end
    % - - - - - - - - - - - - - - - - - - - - - - - - - - - - - - - - - - -
    %> Build the interpolating clothoid arc fixing initial position angle and curvature
    %>
    %> **Usage:**
    %>
    %> \rst
    %> .. code-block:: matlab
    %>
    %>    ok = ref.build_forward( x0, y0, theta0, k0, x1, y1 );
    %>
    %> \endrst
    %>
    %> **On input:**
    %>
    %> - `x0`, `y0`: coordinate of initial point
    %> - `theta0`:   orientation of the clothoid at initial point
    %> - `k0`:       curvature of the clothoid at initial point
    %> - `x1`, `y1`: coordinate of final point
    %>
    %> **On output:**
    %>
    %> - `ok`: true iff the interpolation was successful
    %>
    function ok = build_forward( self, x0, y0, theta0, k0, x1, y1 )
      ok = ClothoidCurveMexWrapper( ...
        'build_forward', self.objectHandle, x0, y0, theta0, k0, x1, y1 ...
      );
    end
    % - - - - - - - - - - - - - - - - - - - - - - - - - - - - - - - - - - -
    %> Find the closest point to a clothoid curve using the
    %> algorithm described in
    %>
    %>  \rst
    %>  | *E. Bertolazzi, M. Frego*, **Point-Clothoid Distance and Projection Computation**,
    %>  | SIAM Journal on Scientific Computing, 2019, 41(5).
    %>  | https://doi.org/10.1137/18M1200439
    %>  \endrst
    %>
    %> **Usage:**
    %>
    %> \rst
    %> .. code-block:: matlab
    %>
    %>    [ X, Y, S, DST ] = ref.closestPoint( qx, qy );
    %>
    %> \endrst
    %>
    %> **On input:**
    %>
    %> - `qx`, `qx`: coordinate of the point
    %>
    %> **On output:**
    %>
    %> - `X`, `Y` : coordinate of the pojected point
    %> - `S`      : curvilinear coordinate along the clothoid of the projection
    %> - `DST`    : point clothoid distance
    %>
    function [ X, Y, S, DST ] = closestPoint( self, qx, qy )
      [ X, Y, S, ~, ~, DST ] =  ClothoidCurveMexWrapper( ...
        'closestPoint', self.objectHandle, qx, qy ...
      );
    end
    % - - - - - - - - - - - - - - - - - - - - - - - - - - - - - - - - - - -
    %> Find the closest point to a clothoid by sampling points
    %>
    %> \rst
    %> .. code-block:: matlab
    %>
    %>    [ X, Y, S, DST ] = ref.closestPointBySample( qx, qy, ds );
    %>
    %> \endrst
    %>
    %> **On input:**
    %>
    %> - `qx`, `qx`: coordinate of the point
    %> - `ds`:       sampling distance  coordinate of the point
    %>
    %> **On output:**
    %>
    %> - `X`, `Y` : coordinate of the pojected point
    %> - `S`      : curvilinear coordinate along the clothoid of the projection
    %> - `DST`    : point clothoid distance
    %>
    function [ X, Y, S, DST ] = closestPointBySample( self, qx, qy, ds )
      [ X, Y, S, DST ] = ClothoidCurveMexWrapper( ...
        'closestPointBySample', self.objectHandle, qx, qy, ds ...
      );
    end
    % - - - - - - - - - - - - - - - - - - - - - - - - - - - - - - - - - - -
    %> Find the distance between a point and a clothoid by sampling
    %>
    %> \rst
    %> .. code-block:: matlab
    %>
    %>    [ DST, S ] = ref.distanceBySample( qx, qy, ds );
    %>
    %> \endrst
    %>
    %> **On input:**
    %>
    %> - `qx`, `qx`: coordinate of the point
    %> - `ds`:       sampling distance  coordinate of the point
    %>
    %> **On output:**
    %>
    %> - `S`   : curvilinear coordinate along the clothoid of the projection
    %> - `DST` : point clothoid distance
    %>
    function [ DST, S ] = distanceBySample( self, qx, qy, ds )
      [ DST, S ] = ClothoidCurveMexWrapper( ...
        'distanceBySample', self.objectHandle, qx, qy, ds ...
      );
    end
    % - - - - - - - - - - - - - - - - - - - - - - - - - - - - - - - - - - -
    %> Return curvature derivatve of the clothoid
    %>
    %> **Usage:**
    %>
    %> \rst
    %> .. code-block:: matlab
    %>
    %>    dk = ref.dkappa(s0,L);
    %>
    %> \endrst
    %>
    function res = dkappa( self )
      res = ClothoidCurveMexWrapper( 'dkappa', self.objectHandle );
    end
    % - - - - - - - - - - - - - - - - - - - - - - - - - - - - - - - - - - -
    %> change the origin of the clothoid curve to curviliear corrdinate `s0`
    %>
    %> **Usage:**
    %>
    %> \rst
    %> .. code-block:: matlab
    %>
    %>    ref.changeOrigin(s0,L);
    %>
    %> \endrst
    %>
    %> **On input:**
    %>
    %> - `s0`: curvilinear coordinate of the origin of the new curve
    %> - `L`:  nel length of the curve
    %>
    function changeCurvilinearOrigin( self, s0, L )
      ClothoidCurveMexWrapper( ...
        'changeCurvilinearOrigin', self.objectHandle, s0, L ...
      );
    end
    %% - - - - - - - - - - - - - - - - - - - - - - - - - - - - - - - - - - -
    %function [ s1, s2 ] = intersect( self, C, varargin )
    %  [ s1, s2 ] = ClothoidCurveMexWrapper( ...
    %    'intersect', self.objectHandle, C.obj_handle(), C.is_type(), varargin{:} ...
    %  );
    %end
    % - - - - - - - - - - - - - - - - - - - - - - - - - - - - - - - - - - -
    %> point at infinity
    %>
    %> **Usage:**
    %>
    %> \rst
    %> .. code-block:: matlab
    %>
    %>    [xp,yp,xm,ym] = ref.infinity();
    %>
    %> \endrst
    %>
    %> - `xp`, `yp`: point at infinity (positive arc)
    %> - `xm`, `ym`: point at infinity (negative arc)
    %>
    function [xp,yp,xm,ym] = infinity( self )
      [xp,yp,xm,ym] = ClothoidCurveMexWrapper( 'infinity', self.objectHandle );
    end
    % - - - - - - - - - - - - - - - - - - - - - - - - - - - - - - - - - - -
    %> Get clothoid parameters
    %>
    %> **Usage:**
    %>
    %> \rst
    %> .. code-block:: matlab
    %>
    %>    [ x0, y0, theta0, k0, dk, L ] = ref.getPars();
    %>
    %> \endrst
    %>
    %> - `x0`, `y0`: initial point of the clothoid arc
    %> - `theta0`:   initial angle of the clothoid arc
    %> - `kappa0`:   initial curvature of the clothoid arc
    %> - `dk`:       curvature derivative
    %> - `L`:        length of the clothoid arc
    %>
    function [ x0, y0, theta0, k0, dk, L ] = getPars( self )
      x0     = self.xBegin();
      y0     = self.yBegin();
      theta0 = self.thetaBegin();
      k0     = self.kappaBegin();
      dk     = self.kappa_D(0);
      L      = self.length();
    end
    % - - - - - - - - - - - - - - - - - - - - - - - - - - - - - - - - - - - - -
    %> Get an optimized sampling of curviliear coordinates on the clothoid arc
    %>
    %> **Usage:**
    %>
    %> \rst
    %> .. code-block:: matlab
    %>
    %>    S = ref.optimized_sample(npts,max_angle,offs);
    %>    S = ref.optimized_sample(npts,max_angle,offs,'ISO');
    %>    S = ref.optimized_sample(npts,max_angle,offs,'SAE');
    %>
    %> \endrst
    %>
    %> **Input:**
    %>
    %> - `npts`:      total number of sampling points
    %> - `max_angle`: initial angle of the clothoid arc
    %> - `offs`:      offset of the curve
    %> - `ISO`:       use ISO orientation of the normal for offset
    %> - `SAE`:       use SAE orientation of the normal for offset
    %>
    %> **Output:**
    %> - `S`: the vector with the sampled curvilinear coordinates
    %>
    function s = optimized_sample( self, npts, max_angle, offs )
      s = ClothoidCurveMexWrapper( ...
        'optimized_sample', self.objectHandle, npts, max_angle, offs ...
      );
    end
    % - - - - - - - - - - - - - - - - - - - - - - - - - - - - - - - - - - -
    %> Plot the clothoid arc
    %>
    %> **Usage:**
    %>
    %> \rst
    %> .. code-block:: matlab
    %>
    %>   ref.plot();
    %>   ref.plot( npts );
    %>   ref.plot( npts, 'Color','blue','Linewidth',2);
    %>
    %> \endrst
    %>
    %> - `npts`: number of sampling points for plotting
    %>
    function plot( self, npts, varargin )
      if nargin < 2
        npts = 1000;
      end
      S = self.optimized_sample( npts, pi/180, 0 );
      [ X, Y ] = ClothoidCurveMexWrapper( 'eval', self.objectHandle, S );
      plot( X, Y, varargin{:} );
    end
    % - - - - - - - - - - - - - - - - - - - - - - - - - - - - - - - - - - -
    %> Plot the clothoid arc with offset
    %>
    %> **Usage:**
    %>
    %> \rst
    %> .. code-block:: matlab
    %>
    %>   ref.plot_offs( offs );
    %>   ref.plot_offs( offs, npts );
    %>   ref.pplot_offslot( offs, npts, 'Color','blue','Linewidth',2);
    %>
    %> \endrst
    %>
    %> - `npts`: number of sampling points for plotting
    %> - `offs`:      offset of the curve
    %>
    function plot_offs( self, offs, npts, varargin )
      S = self.optimized_sample( npts, pi/180, offs );
      [ X, Y ] = ClothoidCurveMexWrapper( 'eval', self.objectHandle, S, offs );
      plot( X, Y, varargin{:} );
    end
    % - - - - - - - - - - - - - - - - - - - - - - - - - - - - - - - - - - -
    %> Plot the curvature of the clothoid curve
    %>
    %> **Usage:**
    %>
    %> \rst
    %> .. code-block:: matlab
    %>
    %>   ref.plotCurvature( npts );
    %>   ref.plotCurvature( npts, 'Color','blue','Linewidth',2);
    %>
    %> \endrst
    %>
    %> - `npts`: number of sampling points for plotting
    %>
    function plotCurvature( self, npts, varargin )
      if nargin < 2
        npts = 1000;
      end
      L = ClothoidCurveMexWrapper( 'length', self.objectHandle );
      S = 0:L/npts:L;
      [ ~, ~, ~, kappa ] = ...
        ClothoidCurveMexWrapper( 'evaluate', self.objectHandle, S );
      plot( S, kappa, varargin{:} );
    end
    % - - - - - - - - - - - - - - - - - - - - - - - - - - - - - - - - - - -
    %> Plot the angle of the clothoid curve
    %>
    %> **Usage:**
    %>
    %> \rst
    %> .. code-block:: matlab
    %>
    %>   ref.plotAngle( npts );
    %>   ref.plotAngle( npts, 'Color','blue','Linewidth',2);
    %>
    %> \endrst
    %>
    %> - `npts`: number of sampling points for plotting
    %>
    function plotAngle( self, npts, varargin )
      if nargin < 2
        npts = 1000;
      end
      L = ClothoidCurveMexWrapper( 'length', self.objectHandle );
      S = 0:L/npts:L;
      [ ~, ~, theta, ~ ] = ...
        ClothoidCurveMexWrapper( 'evaluate', self.objectHandle, S );
      plot( S, theta, varargin{:} );
    end
    % - - - - - - - - - - - - - - - - - - - - - - - - - - - - - - - - - - -
    %> Plot the normal of the clothoid curve
    %>
    %> **Usage:**
    %>
    %> \rst
    %> .. code-block:: matlab
    %>
    %>   ref.plotNormal( step, len );
    %>
    %> \endrst
    %>
    %> - `step`: number of sampling normals
    %> - `len`:  length of the plotted normal
    %>
    function plotNormal( self, step, len )
      for s=0:step:self.length()
        [ x, y, theta, ~ ] = self.evaluate(s);
        n = [sin(theta),-cos(theta)];
        A = [x,x+len*n(1)];
        B = [y,y+len*n(2)];
        plot(A,B);
      end
    end
  end
end
```

### Quick search

### Table of Contents

- Matlab Interface Manual
- C++ API
- MATLAB API

«
hide menu

menu
sidebar
»

### Navigation

- index
- toc
- Clothoids »
- Program Listing for File ClothoidCurve.m

© Copyright 2021, Enrico Bertolazzi and Marco Frego.
Created using Sphinx 4.2.0.
